# Rats exhibit age-related mosaic loss of chromosome Y

**DOI:** 10.1038/s42003-021-02936-y

**Published:** 2021-12-21

**Authors:** Alberto H. Orta, Stephen J. Bush, Mariana Gutiérrez-Mariscal, Susana Castro-Obregón, Lorraine Jaimes-Hoy, Ricardo Grande, Gloria Vázquez, Elisa Gorostieta-Salas, Mónica Martínez-Pacheco, Karina Díaz-Barba, Paola Cornejo-Páramo, Alejandro Sanchez-Flores, Tamas Székely, Araxi O. Urrutia, Diego Cortez

**Affiliations:** 1 Centro de Ciencias Genómicas, UNAM, CP62210 Cuernavaca, México; 2 Weatherall Institute of Molecular Medicine, University of Oxford, OX3 9DS Oxford, UK; 3 Instituto de Biotecnología, UNAM, CP62210 Cuernavaca, México; 4 Instituto de Fisiología Celular, UNAM, Ciudad Universitaria, CP04510 Ciudad de México, México; 5 Unidad Universitaria de Secuenciación Masiva y Bioinformática, Instituto de Biotecnología, UNAM, CP62210 Cuernavaca, México; 6 Laboratorio de Biología Celular y Molecular, Facultad de Ciencias Naturales, Universidad Autónoma de Querétaro, CP76010 Querétaro, México; 7 Instituto de Ecología, UNAM, Ciudad Universitaria, CP04510 Ciudad de México, México; 8 Milner Centre for Evolution, Department of Biology and Biochemistry, University of Bath, BA2 7AY Bath, UK; 9 Department of Evolutionary Zoology and Human Biology, University of Debrecen, H-4032 Debrecen, Hungary

**Keywords:** Genomics, DNA sequencing, Genomic instability

## Abstract

Mosaic loss of the Y chromosome (LOY) is the most frequent chromosomal aberration in aging men and is strongly correlated with mortality and disease. To date, studies of LOY have only been performed in humans, and so it is unclear whether LOY is a natural consequence of our relatively long lifespan or due to exposure to human-specific external stressors. Here, we explored whether LOY could be detected in rats. We applied a locus-specific PCR and target sequencing approach that we used as a proxy to estimate LOY in 339 samples covering eleven tissues from young and old individuals. We detected LOY in four tissues of older rats. To confirm the results from the PCR screening, we re-sequenced 60 full genomes from old rats, which revealed that the Y chromosome is the sole chromosome with low copy numbers. Finally, our results suggest that LOY is associated with other structural aberrations on the Y chromosome and possibly linked to the mosaic loss of the X chromosome. This is the first report, to our knowledge, demonstrating that the patterns of LOY observed in aging men are also present in a rodent, and conclude that LOY may be a natural process in placental mammals.

## Abstract (web summary)

Alberto Orta et al. demonstrate that frequent mosaic loss of chromosome Y (LOY) observed in aging men might also be present in rats. They show that, like in humans, LOY in rats is associated with aging, but is not dependent on environmental stressors, and may be a natural process in placental mammals.

## Introduction

The Y chromosome (ChrY) in placental mammals is known to have lost over 90% of its original genetic material since it originated ~180 million years ago (ma)^1^ from a typical autosome^2^. Since then, ChrY retained only a small number of genes, long thought to be mainly involved in functions related to spermatogenesis and sex determination^3,4^.

Past studies have found that of all the somatic chromosomic aberrations that arise in aging men, mosaic loss of chromosome Y (LOY) is the most frequent one^5,6^. In large association studies, where thousands of adult men have been analyzed using SNP-arrays^6,7^, the frequency of LOY in peripheral blood cells in men between 37–73 years was reported to be as high as 16–20% on average^7–10^, with men of 40 years having 2.5% of LOY, whereas men of 70 years showed 43.6% of LOY^7^ and men of 93 years presented 57% of LOY in >10% of their nucleated blood cells^11^. More recently, a study showed that LOY was detectable in saliva at a rate of 5.14% in men of 70–74 years, 17.4% in men of 80–84 years, and 42.9% in men of 90–93 years^12^. Increased frequencies of LOY could be related to clonal expansion^13^.

LOY in nucleated blood cells of aged men has been linked to increased risk of mortality^5,11,14,15^. LOY has been associated with the increased prevalence of several diseases including several types of cancer^5,10,16,17^, macular degeneration^18,19^, Alzheimer’s disease^20^, cardiovascular diseases^21,22^, type 2 diabetes^10^ as well as obesity^10^ and harmful behaviors such as suicide completion^23^. The results from these large association studies suggest that genes found on ChrY could be involved in important cellular processes besides spermatogenesis and sex determination^3,4^, as previously proposed^1,24^.

In addition to blood cells, LOY is also detectable in brain^23^ and buccal mucosa^6,11^, and has been inferred in liver cells^25,26^. LOY does not affect tissues equally and has been found to be more common in blood cells than in the brain^27^. Interestingly, a recent study found that LOY affects cell types at different frequencies in a disease-specific manner. For example, in Alzheimer’s disease, LOY is more frequent in natural killer cells, whereas in prostate cancer LOY is more frequent in CD4+ T-lymphocytes^28^. These findings suggest that LOY does not follow a systemic pattern affecting all cell types in the same way and that specific diseases can be linked to specific LOY profiles in different cell types.

As LOY is most often identified in men aged 50 years or older^7^, it is possible that LOY could be a consequence of the evolution of an unusually long lifespan in humans (AnAge Database of Animal Ageing and Longevity, https://genomics.senescence.info/species/index.html)^29^. It is also possible that LOY may be triggered by human-specific stressors or lifestyle factors, such as smoking^30^, outdoor air pollution^22^, exposure to insecticides^31^, and heavy drinking^10^.

Studying LOY in other species could provide important clues to understanding its origins and evolution^32^. However, to date, LOY remains understudied in non-human species. In blood samples obtained from 25 bulls for over 30 months, copy number variations in the Y-linked *TSPY* gene have been found to both increase and decrease with age^33^. Moreover, by studying eight old mice, aneuploidies of chromosomes 7, 8, and Y were found to accumulate with age in the cerebral cortex using in situ hybridization and flow cytometric analysis and sorting (FACS)^34^. However, due to limitations of the techniques used or small sample sizes, it is unclear whether LOY is a general pattern in these species. Thus, conclusive evidence of LOY in non-human species is lacking. In this work, we explored whether LOY is present in the rat under laboratory conditions.

## Results

### PCR screenings of 339 samples from eleven tissues indicate LOY is acquired during aging in rats

We estimated the frequency of LOY in 339 samples from eleven tissues (blood, brain, heart, kidney, liver, lung, muscle, pancreas, skin, spleen, and testis) retrieved from young (three months old; 134 samples) and old (22–25 months old; 205 samples) male rats. To do so, we applied a method based on a locus-specific PCR and target sequencing approach that allowed us to quantify the copy number of single-copy genes *EIF2S3Y* (on chromosome Y) and *COL1A1* (autosomal gene, located on chromosome 10) (see Methods). We selected *EIF2S3Y* because this gene has no pseudogene copies on the reference genome that could affect copy number estimates. The relative difference in coverage between these two genes was used as a proxy for LOY (see Methods).

**Fig. 1:**
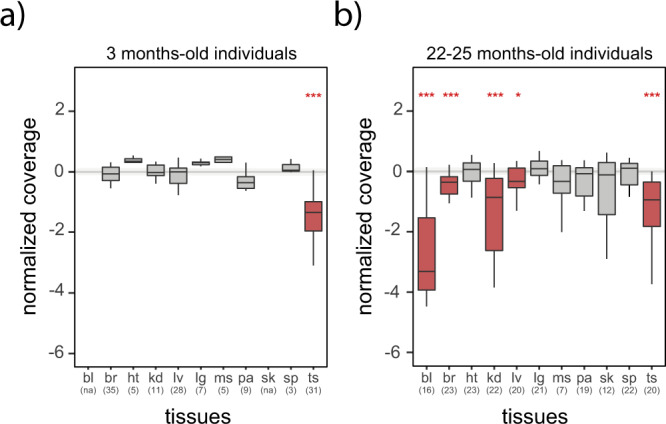
Normalized copy number of *EIF2S3Y* in eleven tissues from young and old rats. **a** Coverage of *EIF2S3Y* normalized by the coverage of the autosomal gene *COL1A1* in nine tissues of three months old rats (data from blood and skin were inadvertently lost; see Methods). **b** Coverage of *EIF2S3Y* normalized by the coverage of the autosomal gene *COL1A1* in eleven tissues of 22–25 months old rats. **a**, **b** bl is blood, br is brain, ht is heart, kd is kidney, lv is liver, lg is lung, ms is muscle, pa is pancreas, sk is skin, sp is spleen, ts is testis. Significant differences, Mann–Whitney *U* test against a distribution with a fixed median of 0 (no difference against expected estimates). *P* values were Benjamin–Hochberg corrected: * indicates *P* < 0.05; *** indicates *P* < 0.001. Sample sizes are indicated in parenthesis. Error bars, maximum and minimum values, excluding outliers.

In young rats, we observed significant levels of LOY only in one tissue, the testis (Fig. 1a; Mann–Whitney *U* test against a distribution with a fixed median of 0, *P* < 0.05). Testis showed low *EIF2S3Y* copy numbers relative to the autosomal gene, consistent with a high number of haploid cells in this tissue. In contrast, old rats showed significant LOY in blood, brain, kidney, liver, and testis (Fig. 1b; Mann–Whitney *U* test against a distribution with a fixed median of 0, *P* < 0.05). No significant levels of LOY were identified in heart, lung, muscle, pancreas, skin, and spleen (Fig. 1b). We found no difference in the testis between young and old rats (Fig. 1a, b; Mann–Whitney *U* test, *P* < 0.05), meaning the observed LOY in younger rats did not increase over time. LOY differences in brain, kidney, and liver between young and old rats were significant (Fig. 1a, b; Mann–Whitney *U* test, *P* < 0.05), indicating that in these three tissues LOY increases with aging.

### Re-sequencing of 60 genomes confirm LOY in old rats

The PCR-based method examined a single locus, the *EIF2S3Y* gene, as a proxy for LOY. We reasoned, however, that PCR stochasticity and/or microdeletions on the *EIF2S3Y* locus could affect LOY estimates. To confirm that LOY was present in rats, we selected 60 samples for whole-genome re-sequencing; samples belonged to old rats, comprised the eleven tissues, and had a greater likelihood of having LOY based on the PCR screening (Supplementary Fig. 1). We also analyzed the genome of a three months old male rat as a negative control.

**Fig. 2:**
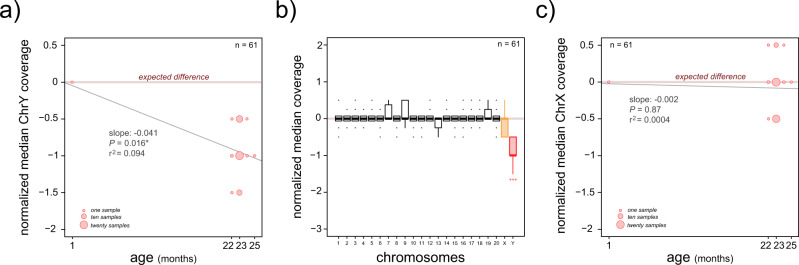
Normalized sequencing coverage of full Y and X chromosomes in samples from young and old rats. **a** Dot plot of the normalized median sequencing coverage of ChrY compared to the age of the rats. The observed sequencing coverage of the Y and X chromosomes was standardized using an expected value calculated as half of the median autosomal sequencing coverage (both sex chromosomes are single copy in the male genome). Significant differences, Linear Model: lm[median.chrY.coverage ~ age], * indicates *P* < 0.05. **b** Boxplot of the normalized median sequencing coverage for the 22 chromosomes in rats (20 autosomes and two sex chromosomes). The observed coverage for each autosome was normalized by the median coverage across autosomes. Only half of the autosomal coverage was used to normalize values from Y and X chromosomes. Significant differences, Mann–Whitney *U* test against a distribution with a fixed median of 0 (similar to the expected value). *P* values were Benjamin–Hochberg corrected: *** indicates *P* < 0.001. Error bars, maximum and minimum values, excluding outliers. **c** Dot plot of the normalized median sequencing coverage of chromosome X compared to the age of the rats. The observed coverage of the Y and X chromosomes was standardized using half of the median autosomal coverage. Significant differences, Linear Model: lm[median.chrX.coverage ~ age]. Sample sizes are indicated in each plot (n value).

We calculated the difference between the observed data (ChrY median sequencing coverage) and compared it to the expected estimate, which was half of the autosomal sequencing coverage given that the Y chromosome is present as a single copy in the genome. We found that samples from older rats (22–25 months old) showed lower than expected ChrY median coverage (Fig. 2a). This result confirms that LOY is present in rats. Coverage estimates from the PCR-based method and the whole-genome re-sequencing data were positively correlated (Supplementary Fig. 2).

Some samples appeared to show mosaic loss of the X chromosome (also present as a single copy in a male genome) (Fig. 2b, c) and we found a significant association between the median coverage of Y and X chromosomes (Linear Model: lm[median.chrY.coverage ~ median.chrX.coverage], slope = 0.41, *P* = 0.0008), which it is likely due to samples with high LOY frequency also showing some X chromosome loss (Supplementary Fig. 3). Overall, however, no significant loss of the X chromosome was observed (Fig. 2b, c) and of the 22 chromosomes present in rats, the Y chromosome is the only one to show significantly low coverage (Mann–Whitney *U* test against a distribution with a fixed median of 0; Fig. 2b).

Comparison across tissues did not reveal significant differences in LOY between the eleven tissues that were re-sequenced (Kruskal–Wallis rank-sum test, *P* < 0.05). So, although LOY may have tissue-specific occurrences (Fig. 1b), it is present in many tissues in aged rats. The origin of LOY has been hypothesized to come from chromosomal segregation setbacks during mitosis^32^. Thus, we reasoned that tissues with higher rates of cell division could also have higher rates of LOY. We decided to test this hypothesis by comparing LOY estimates against data from eleven tissues covering cellular division rates, cellular longevities, and the length of telomeres. We did not find a significant association between LOY and these three traits (Spearman’s rank correlation rho, *P* < 0.05; Supplementary Fig. 4; see Supplementary Data 1 for data and references).

### Coverage analysis over the Y chromosome sequence revealed variations in LOY

**Fig. 3:**
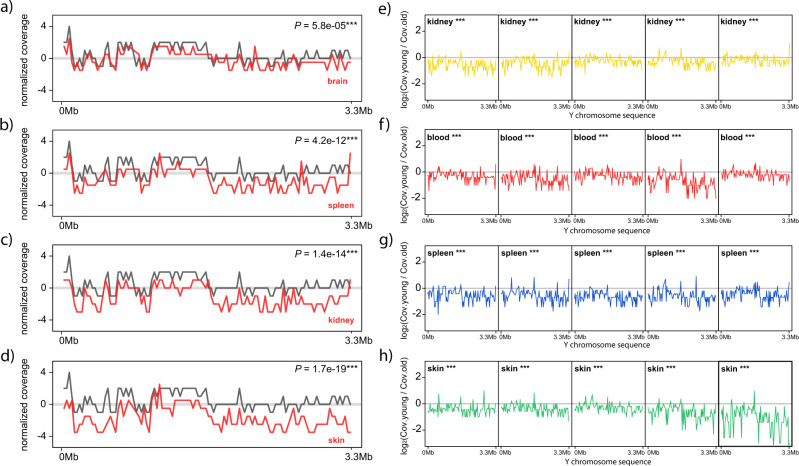
Normalized sequencing coverage along the ChrY in samples from a young rat and old rats. **a** Normalized sequencing coverage from a young rat (grey line) and the normalized sequencing coverage from a brain sample with minor LOY (red line). **b** Same as in (**a**) but showing the normalized sequencing coverage from a spleen sample with medium LOY (red line). **c** Same as in (**a**) but showing the normalized sequencing coverage from a kidney sample with marked LOY (red line). **d** Same as in a) but showing the normalized sequencing coverage from a skin sample with marked LOY (red line). **a**–**d** Significant differences, Mann–Whitney *U* test between the coverage of the young and old samples. *P* values were Benjamin–Hochberg corrected. Exact *P* values are indicated. The grey line indicates the expected coverage in the absence of LOY. **e**–**h** Log2 ratios of the sequencing coverage in the young rat (Cov.young) divided by the sequencing coverage in old rats (Cov.old) using non-overlapping sliding windows of 100 kb over the first 3.3 Mb of the Y chromosome sequence. The grey line indicates a log2 ratio of zero, which represents an identical coverage between the young and old rats. Log2 ratios above zero may indicate duplicated regions in the re-sequenced genomes that are not reported in the reference genomic sequence. Log2 ratios below zero may indicate loss of the Y chromosome in the re-sequenced genomes. Five samples from kidney are shown in yellow; five samples from blood are shown in red; five samples from spleen are shown in blue; five samples from skin are shown in green. Significant differences, Mann–Whitney *U* test against a distribution with a fixed median of 0 (similar sequencing coverage between the young and old samples). *P* values were Benjamin–Hochberg corrected: *** indicates *P* < 0.001.

We examined read coverage along the sequence of the Y chromosome to reveal potential patterns owing to partial losses. To directly compare coverage variations between samples, we standardized the samples using their sequencing coverage of uniquely mapped reads. The three months old rat showed some coverage variations along the ChrY (Fig. 3a–d, grey line), although the overall coverage was not significantly different from the expected value (half of the autosomal coverage; Mann–Whitney *U* test against a distribution with a fixed median of 0, *P* < 0.05). In samples from old rats, we found that ChrY mirrored the coverage variations observed in the young rat, although their coverage values were consistently lower (Fig. 3a–d, red lines). We think differences in coverage along the ChrY sequence may be the result of unresolved structures in the current Y chromosome sequence draft; sex chromosomes are particularly rich in repeated elements^35^ but the reference genome may contain only one copy of what is actually a complex repeat, resulting in coverage variations. We show in Fig. 3 an example with minor LOY (Fig. 3a, red line), an example with medium LOY (Fig. 3b, red line), and two examples with marked LOY (Fig. 3c, d, red lines).

**Fig. 4:**
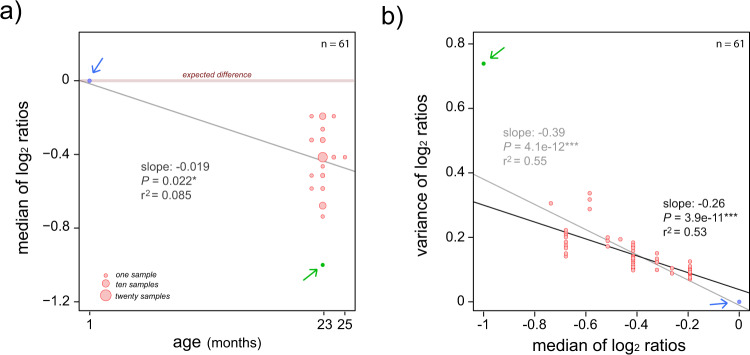
Log_2_ ratios of ChrY coverage compared to the age of the rats. **a** Dot plot showing the median values of the log_2_ ratios of ChrY sequencing coverage in young versus old rats. The blue point at value zero represents the log_2_ ratio of ChrY from the young rat against itself. The green dot is the sample from old rats with the greatest coverage difference against ChrY from the young rat (Fig. 3h, fifth sample). The arrows point at these values. Significant differences, Linear Model: lm[median.chrY.ratios ~ age]. Exact *p* values are indicated, * represents *P* < 0.05. **b** Dot plot of the median values of the log_2_ ratios against its variance. Significant differences, Linear Model: lm[variance.ratios ~ median.chrY.ratios]. Exact *p* values are indicated, * represents *P* < 0.05; *** represents *P* < 0.001. The results from two linear models are shown: the linear tendency in light grey includes all data points, whereas the linear tendency in black excluded the blue dot (young ChrY) and the green dot (ChrY with the lowest log_2_ ratio). The arrows point at these values.

Next, we contrasted the coverage along the sequence of ChrY between the young rat and each of the ChrY from old rats. To do so, we calculated the log_2_ ratio of ChrY coverage in the young rat divided by the ChrY coverage in every sample from old rats (Fig. 3e–h; Supplementary Figs 5–8). Similar coverage between young and old samples would result in ratios close to zero. In contrast, as LOY increases, the log_2_ ratios would become more negative. We found that the log_2_ ratios recapitulated the presence of LOY in the eleven tissues of old rats, both by comparing the median values of the log_2_ ratios against the age of the rats (Fig. 4a) or by examining the individual samples where the log_2_ ratios along the ChrY sequence are generally below zero (Fig. 3e–h; Supplementary Figs. 5–8).

We noticed that some samples showed rather smooth log_2_ ratios over the ChrY sequence (Fig. 3e, 5th kidney sample; Fig. 3f, 5th blood sample; Fig. 3h, 3rd skin sample; further examples in Supplementary Figs. 5–8), whereas other samples showed larger variations (Fig. 3e, 1st kidney sample; Fig. 3f, 4th blood sample; Fig. 3g, 4th spleen sample; Figs. 3h, 5th skin sample; further examples in Supplementary Fig. 5–8). We calculated the variance of the log_2_ ratios per sample and found a significant negative correlation against the median value of the log_2_ ratios (Fig. 4b). We observed a similar pattern when we computed the variance of ChrY coverage against the median of ChrY coverage (Supplementary Fig. 9). These results suggest that LOY is strongly associated with other types of structural aberrations on the Y chromosome.

## Discussion

To the best of our knowledge, this study represents the first conclusive evidence of mosaic loss of the Y chromosome in another placental mammal besides humans. We worked with rats because we could be confident about the age of the individuals, something incredibly difficult when working with species in the wild. Laboratory rats live about 2–3.5 years^36,37^ with reproductive senescence occurring between 15 and 20 months of age^37^. In this work, we compared the copy number of the Y chromosome in young rats against senescent rats. We found that, as in humans, the frequency of LOY was higher in older individuals. Our results indicate that both short-lived and long-lived species have similar patterns, suggesting LOY is a natural consequence of the aging process in placental mammals.

Our data also suggests that LOY is likely associated with other ChrY structural aberrations and probably loss of the X chromosome, although a larger sample size would be needed to corroborate the latter. In humans, the X chromosome is also lost during aging^15^, particularly the inactivated X chromosome^38^. Although recent data suggest that gains of the X chromosome are more tolerable than the mosaic loss of this sex chromosome in peripheral leukocytes of men^39^.

The PCR-based approach uses high-throughput sequencing data to compare the coverage of a single-copy ChrY gene against the coverage of a single-copy autosomal gene as a proxy for LOY. We noticed that LOY estimates from this approach showed larger variations compared to estimates from whole-genome re-sequencing data; the PCR-based method is more susceptible to some stochasticity whereas whole-genome data allows for multiple measurements along the Y chromosome that are then summarized using a median value. We would like to highlight two important results regarding the methods: (1) both the genomic data and the PCR-based data coincided that the copy number of the Y chromosome was lower than expected in old rats; and (2) the significant positive correlation between the two methods indicated that, for most cases, lower coverage values in the genomic data were also associated with lower coverage values in the PCR data, and vice-versa. We acknowledge that the PCR-based method could serve as an initial approach to test for LOY in a species/tissue at the reasonable price of 80 USD per sample using medium/large sample sizes. We discourage, however, taking the values for individual samples from the PCR-based method as exact LOY estimates; more precise LOY estimates for specific samples should come from whole-genome sequencing data.

Most studies about LOY have examined peripheral blood cells in humans. In this study, we were able to examine eleven tissues of a non-human mammal, contributing to a more extended knowledge of LOY across mammals. We found that while all tissues were susceptible to LOY, overall, blood, kidney, brain, and liver may be more affected by this process. Remarkably, blood, brain, and liver are three tissues where LOY has been detected or inferred in humans^11,25–27,32^. Possibly, mosaic loss of the Y chromosome has a direct effect on the functioning of cells as seen in the blood^40^. We found that differences in the frequency of LOY between tissues in the PCR approach were no longer significant when we examined the data from whole-genomes; we noted that this result may seem contradictory. However, the result is perfectly normal since we only sequenced the genomes of those samples where LOY was more likely to occur based on the PCR method. Moreover, we limited the number of samples per tissue to five, thus greatly reducing the statistical power for any across-tissue comparison.

One interesting hypothesis suggested that the origin of LOY could be related to chromosomal segregation setbacks during mitosis^32^. So, we reasoned that tissues with elevated rates of cell division, such as blood and skin, would have higher levels of LOY in contrast to tissues like brain and heart that have low rates of cell division. We found no correlation between LOY and rates of cell division in the eleven tissues. Nevertheless, in post-mitotic cells, there are constant genome dynamics that could influence LOY. Particularly, in the brain, it is worth considering that the mosaic nature of the mammalian neuronal genome increases with age (reviewed in^41^) and the potential presence of LOY already during early developmental stages since experiments using Y chromosome-specific fluorescence in situ hybridization (FISH) found that 4.5% of neurons in the embryonic cortex in mice lacked a Y chromosome^42^.

In humans, external environmental stressors have been linked to increased LOY, for example smoking^30^, outdoor air pollution^22^, exposure to insecticides^31^, and heavy drinking^10^. The rats in this study, however, were not subjected to any obvious external environmental stressors that could be considered as confounding forces explaining LOY. We think LOY is an intrinsic feature of the aging process of placental mammals linked to the significant degeneration of their Y chromosome. A refined and more complete sequence of the Y chromosome in rats will allow the analysis of the repeated regions and the detailed detection of structural aberrations on this sex chromosome.

In mammals, females live longer than males and this pattern is strongly associated with the type of sex determination system^43^. LOY has been proposed as a potential force affecting sex-specific survival differences^44^. Finding LOY in a well-known lab model has the potential to accelerate the research about the molecular factors underlying LOY during aging. Our study was designed to establish the presence of LOY in the rat. We expect that our results will motivate future studies of LOY in rodents to establish whether LOY recapitulates other patterns such as the association between LOY and increased mortality and/or its association with exposure to environmental stressors as is the case in humans.

The association between LOY and specific diseases^5,10,14–17^ is likely the result of a disruption in the functions carried out by the Y-linked genes. For example, some Y-linked genes in humans appear to act as tumor suppressors: the *TMSB4Y* gene regulates cell morphology and cell proliferation^45^ and the *KDM5D* gene probably protects against renal and prostate cancer^46,47^. However, the gene content of the Y chromosome in rats and humans is different. Of the 16 ancestral gametologs found in placental mammals, only nine are shared between great apes and rodents^48^. *TMSB4Y*, for example, has been lost in many rodent species, including rats^48^. It is possible, therefore, that while LOY is present in the same tissues in humans and rats, it may not affect the species’ health in a similar way.

## Methods

### Rat husbandry

Wistar rats were obtained from the animal facilities of the Instituto de Biotecnología and the Instituto de Fisiología Celular at the National University of Mexico (UNAM). In both facilities, animals were housed at an ambient temperature of 22 ± 2 °C, relative humidity of 55 ± 15%, and a day/night cycle of 12 h/12 h with food (Teklad Cat. No. 2018SX) and water ad libitum. Animals were housed as follow: from weaning to two months of age, they were kept four animals per cage in standard rat cages (Lab Products, 48.26 cm width, 26.035 cm depth, 20.32 cm height); from two to five months of age, animals were changed to jumbo-size cages (Lab Products, 50.8 cm width, 40.64 cm depth, 20.32 cm height), 3 animals per cage; from five months of age onwards (22–25 months), animals were kept in rabbit cages in groups of two or three animals per cage (Lab Products, 66.04 cm width, 66.04 cm depth, 43.815 cm height). Animals were euthanized using a guillotine. The procedure was performed by an experienced technician. All animal procedures were approved by the Bioethical Committee of the Instituto de Biotecnología (approval number 343) and the Internal Committee of Care and Use of Laboratory Animals of the Instituto de Fisiología Celular (IFC-SCO51-18).

### DNA extraction

For solid tissues, we collected 25 mg of tissue and we purified DNA using the QIAamp Fast DNA Tissue Kit from QIAGEN (Cat. No. 51404) or the Zymo Quick-DNA Microprep Kit (Cat. No. D3024). For blood, we collected 150 µl and we purified DNA using the Blood DNA Isolation Mini Kit from NORGEN BIOTEK CORP (Cat. No. 46300/ 46380). We verified the integrity of the DNA using 1% agarose gels. All DNA samples were tested for integrity (260/280 and 260/230 ratios >1.8), using a NanoDrop 2000 spectrophotometer (Thermo Scientific), and quantified in a Qubit 4 fluorometer (Thermo Scientific, MA, USA) with the Qubit dsDNA BR Assay kit from the same supplier. Some samples were not conserved properly and the samples were lost (blood and skin from young rats).

### *EIF2S3Y* locus-specific PCR, sequencing, and analyzes

We designed the primers using the AmplifX software (v.2.0.7; by Nicolas Jullien, https://inp.univ-amu.fr/en/amplifx-manage-test-and-design-your-primers-for-pcr) based on the reference genome downloaded from the ENSEMBL database (v.98, Rnor_6.01; https://www.ensembl.org/Rattus_norvegicus/). The autosomal primer (i.e. control primer) was designed for chromosome 10, based on the nucleotide sequence of the single-copy gene *COL1A1*. We verified in the ENSEMBL database that this gene (ENSRNOG00000003897) showed no closely related paralogues in the reference genome. We designed the forward primer on exon 36 (5ʹ TGCGAAAGGTGAACCTGGTGAT 3ʹ) and the reverse primer on intron 38 (5ʹ ACTGTCAGAGTCCAAGCTTCCA 3ʹ). We also selected the single-copy gene *EIF2S3Y* (located on the Y chromosome of the rat) to design male-specific primers. The Y-linked gene (ENSRNOG00000060048) has a gametologue on the X chromosome (*EIF2S3X*; ENSRNOG00000060793) that is 98% identical at the CDS level, which could cause undesired co-amplification of the X locus. To increase the specificity for *EIF2S3Y*, we designed the forward primer on exon 11 (5ʹ GCAGTCAAGGCAGATTTGGGTA 3ʹ) and the reverse primer on intron 11 (5ʹ AGCACTCCCAAAGCAATCATAAGG 3ʹ); both primers were unique to *EIF2S3Y* and shared no similarities with the *EIF2S3X* sequence. The designed PCR products for *COL1A1* and *EIF2S3Y* were 550 nucleotides long. We verified the male-specificity of *EIF2S3Y* by standard PCR amplification using five male and five female samples. We used the Phusion Flash High Fidelity from Thermo Fisher Scientific (Cat. No. F548L) with the following program: first 98 °C - 10 s, then 30 cycles of 98 °C - 2 s, 66 °C - 5 s and 72 °C - 10 s, with a final elongation step at 72 °C - 30 s. We verified the size of the PCR products in a 1% agarose gel.

We confirmed that *COL1A1* and *EIF2S3Y* were single-copy in our rats using standard qPCR curves with five samples. We used four DNA dilutions: 0 ng/µl, 0.2 ng/µl, 2 ng/µl, and 20 ng/µl. We selected a Tm of 61 °C and a final concentration of primers of 10 pM. We used the PowerUp SYBR Green Master Mix from Thermo Fisher (Cat. No. A25741) with the following program: one cycle at 50 °C - 2 min, one cycle at 95 °C - 10 min, then 40 cycles at 95 °C - 15 s, 60 °C - 30 s and 72 °C - 30 s, finally, one cycle at 95 °C - 15 s and 60 °C - 30 s. We used a MicroAmp Optical 96-well Reaction plate from Applied Biosystems (Cat. No. 4316813) and a CFX96 Touch Real-Time PCR instrument (Bio-Rad). We calculated the slope of the Ct values from the four serial dilutions and estimated the relationship between *COL1A1* and *EIF2S3Y* as the log_2_ ratio of the slope from *EIF2S3Y* divided by the slope from *COL1A1*.

We re-synthesized the primers for *COL1A1* and *EIF2S3Y* adding two overhangs needed for Illumina library construction: forward overhang 5ʹ TCGTCGGCAGCGTCAGATGTGTATAAGAGACAG‐[locus‐ specific sequence] and reverse overhang 5ʹ GTCTCGTGGGCTCGGAGATGTGTATAAGAGACAG‐[locus‐ specific sequence]. We verified that the patterns of PCR products remained unchanged using the primers with overhangs in 1% agarose gels. DNA samples were standardized to 10 ng/µl. Then, we performed the amplification of *COL1A1* and *EIF2S3Y* in the same reaction using the Phusion Flash High Fidelity from Thermo Fisher Scientific (Cat. No. F548L). We followed the program: one cycle at 98 °C - 10 s, then 30 cycles at 98 °C - 2 s, 58 °C - 5s, and 72 °C - 10 s, with a final elongation step at 72 °C - 30 s. We purified the PCR products using Agencourt AMPure XP from Beckman Coulter (Cat. No. A63882) following the manufacturer’s recommendations (22.5 µl per sample). Purified PCR products were quantified by Qubit 1X dsDNA HS Assay Kits y dsDNA Br Assay Kit from Thermo Fisher (Cat. No. Q33231 and Q32853, respectively). Samples were then multiplexed and sequenced in a NextSeq 500 Illumina machine in the Unidad Universitaria de Secuenciación Masiva y Bioinformática at UNAM. A 150-cycle sequencing kit was used with a pair-end configuration to generate reads 75 nucleotides long.

The quality of the reads was verified using FastQC (http://www.bioinformatics.babraham.ac.uk/projects/fastqc), and the remaining adaptors were removed with Trimmomatic (v.0.36)^49^. Reads from the PCR products were subsequently aligned using bowtie2 (v.2.3.4.1)^50^ against the complete reference genome of the rat downloaded from the ENSEMBL database (v. 98, Rnor_6.01; https://www.ensembl.org/). We used samtools^51^ to extract the unique mapping reads to either *COL1A1* or *EIF2S3Y*. We obtained on average 440,000 reads (SD: ±138,000) per sample that mapped to the two loci (Supplementary Data 2). We log_10_ transformed the read counts for *EIF2S3Y* and *COL1A1* (we divided the read count of *COL1A1* by two before applying a log_10_). We then calculated the difference between the expected value (half of the *COL1A1* sequencing coverage) and the observed value (sequencing coverage from *EIF2S3Y*).

### Full genome re-sequencing and analyzes

We selected 60 samples showing the highest LOY frequency based on the PCR screening, five for every tissue except for the kidney from which we selected ten samples. The 60 DNA samples were standardized to have 3 µg of DNA in 30 µl of TE buffer. Genomic DNA was sequenced at Novogene Facilities in California, USA. We generated on average 30 Gb of data per sample. Reads were paired-end and 150 nucleotides long. We also used a genome from a male rat (SAMN02333824) that we re-sequenced in a previous study^1^. The re-sequenced genome came from the liver of a three months old male rat. The quality of the reads was verified using FastQC (http://www.bioinformatics.babraham.ac.uk/projects/fastqc), and the remaining adaptors and low-quality bases were trimmed with Trimmomatic (v.0.36)^49^. Reads were aligned using bowtie2 (paired-end option; v.2.3.4.1)^50^ with the following parameters: -q–local–very-sensitive-local–all–no-unal to obtain all the alignments for each read. Reads were aligned against the complete reference genome of the rat downloaded from the ENSEMBL database (v. 98, Rnor_6.01; https://www.ensembl.org/Rattus_norvegicus/). We replaced the Y chromosome from ENSEMBL with the Y chromosome from the UCSC database (mRatBN7.2/rn7, released on Nov. 2020; https://genome.ucsc.edu/) because it was recently upgraded and increased the length by an extra 15,005,383 nucleotides (35% of N). Because ChrY has a complex and repeated structure, we worked in all the analyzes with unique mapped reads and we limited the analyses for the Y chromosome to its non-recombining region, also known as the male-specific region. We used samtools (v.1.9)^51^ to select the reads mapping to a single position on the genome and sorted these unique mapping reads according to their position on the 22 chromosomes in the rat genome (20 autosomes and two sex chromosomes). We verified the mapping results of uniquely mapping reads using Blastn (v.2.9.0; parameters: -dust no -evalue 0.00001 -outfmt 6 -num_threads 5)^52^ searches against the complete reference genome of the rat; we requested that reads did not show more than one alignment with >98% identity. Next, for each sample, we calculated the median coverage across the 20 autosomes as a proxy for the expected copy number of chromosomes; average coverage per sample was 11X-13X using all reads and 7X-10X using unique mapping reads (Supplementary Data 3). Then, to perform comparisons against the X and Y chromosomes, we divided the average autosomal coverage by two, since sex chromosomes in males are present in a single copy. Finally, we calculated the median coverage of Y and X chromosomes and then computed the difference between the expected coverage (half of the autosomal coverage) and the observed coverage value of Y and X chromosomes.

Unique mapped reads on the Y chromosome were located in the first 3.3 Mb, where all the protein-coding genes are located too. The rest of ChrY in the rat is composed of repeated elements; repeated DNA is complex and extremely difficult to work with, therefore, coverage analyzes of these regions tend to be poorly accurate due to large estimates when the number and structure of the repeated elements are not well-established, as in the case of rat, or because reads can map to hundreds of positions with equal probability. Thus, the analyzes we performed were made on the first 3.3 Mb of ChrY. To compare values across samples, genome coverages were normalized by the sequencing depth of unique mapped reads in each sample. We then calculated the coverage of unique mapped reads over the sequence of ChrY using non-overlapping sliding windows of 10,000 nucleotides long. For each window, we calculated the log_2_ ratio of the sequencing coverage from the young rat divided by the sequencing coverage from an old rat. We repeated this operation for the 60 re-sequenced genomes. The variance was calculated for each sample using the log_2_ ratios of all windows. For visual purposes, in Fig. 3 and Supplementary Figs. 5–8, we used non-overlapping sliding windows of 100,000 nucleotides.

### Statistics and reproducibility

All statistical analyses were performed using the R package, standard libraries: we performed two-sided Mann–Whitney *U* tests, wilcox.test(), paired = FALSE; Linear Models, lm(y~x), Kruskal–Wallis rank-sum test, Kruskal.test(), and two-sided Spearman’s rank correlation, cor.test, method = “spearman”. We specified the parameter “mu = 0” to compute the Mann–Whitney *U* test against a distribution with a fixed median of 0. Data were plotted using the R package, “ggplot2” library (https://ggplot2.tidyverse.org).

## Supplementary information

Supplemental Information

Description of Additional Supplementary Files

Supplementary Data 1–3

## Data Availability

Data generated for this project are available from the NCBI database (http://www.ncbi.nlm.nih.gov/) under BioProject PRJNA752649. Normalized coverage data and log2 ratios for the 60 genomes can be found trough the Figshare platform: https://figshare.com/articles/dataset/Alberto_Orta_etal_2021_coverage_data_for_60_rat_genomes/16992019. Supplementary Data 1 contains the data for Supplementary Fig. 4. Supplementary Data 2 contains the data for Fig. 1 and Supplementary Fig. 1. Supplementary Data 3 contains the data for Fig. 2, Fig. 4, and Supplementary Figs. 2, 3, and 9. Tables uploaded to the Figshare platform contain the data for Fig. 3 and Supplementary Figs. 5–8.
