# Supplementary material for: Rats exhibit age-related mosaic loss of chromosome Y
Source: Commun Biol. 2021 Dec 21;4:1418. doi: 10.1038/s42003-021-02936-y (PMC8692441; doi:10.1038/s42003-021-02936-y)
Supplement: Supplementary file 1 — Supplemental Information [file 42003_2021_2936_MOESM1_ESM.pdf]

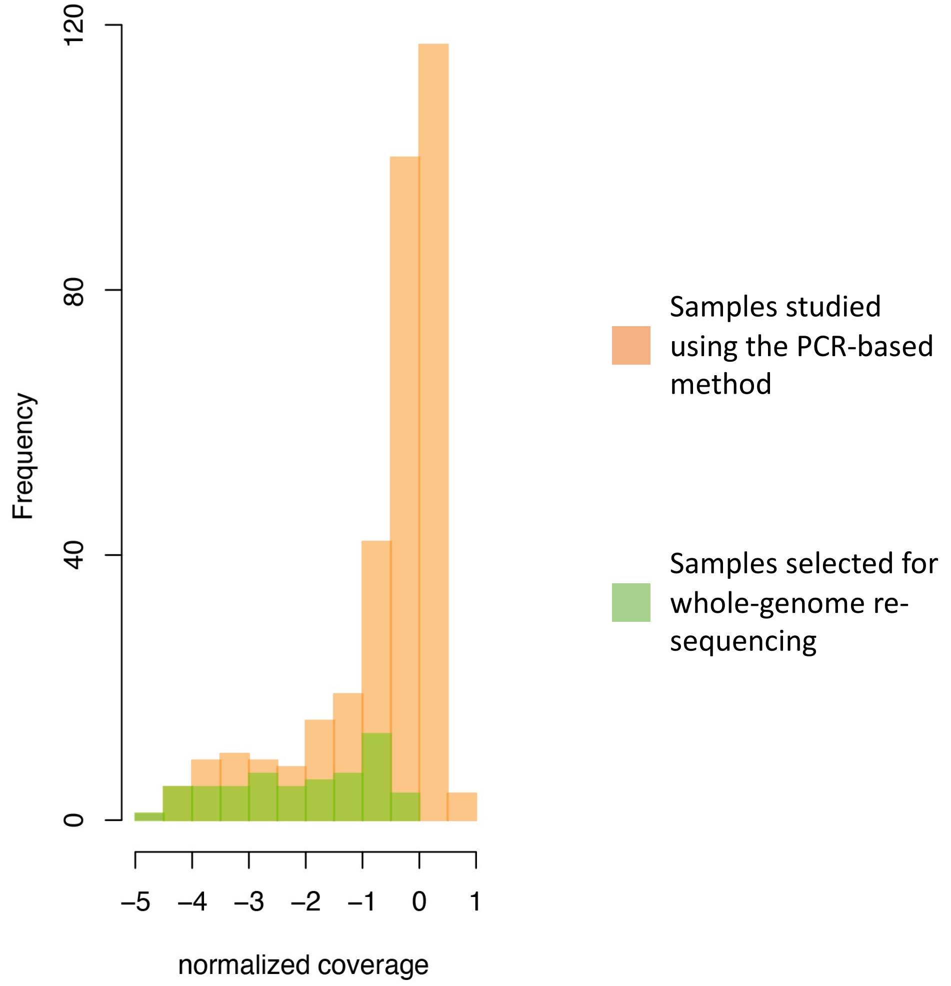

Supplementary Figure 1. Histogram showing the frequency and coverage of samples studied using the PCR-based method (orange) and the 60 samples selected for whole-genome re-sequencing (green); these samples belonged to old rats, comprised the eleven tissues, and had a greater likelihood of having LOY based on the PCR screening.

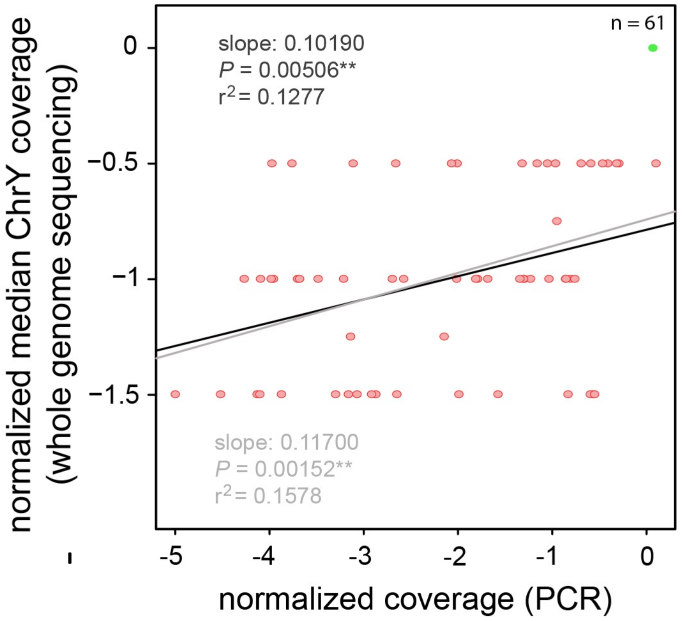

Supplementary Figure 2. Dot plot showing the normalized coverage obtained using the PCR-based method against the normalized median ChrY coverage from the whole-genome re-sequencing data. Significant differences, Linear Model: lm[median.pcr ~ median.chrY.genome]. Exact p-values are indicated, ** represents $P < 0.01$. The results from two linear models are shown: the linear tendency in light grey includes all data points, whereas the linear tendency in black excluded the green dot (young ChrY). We think there are two important messages in this figure: 1) both the genomic data and the PCR-based data coincided that the copy number of the Y chromosome was lower than expected in old rats; and 2) the significant positive correlation between the two methods indicated that, for most cases, lower coverage values in the genomic data were also associated with lower coverage values in the PCR data, and vice-versa. We observed that LOY estimates from the PCR-based method showed larger variations compared to estimates from whole-genome re-sequencing data; whole-genome data allows for multiple measurements along the Y chromosome that were summarized using a median value (medians are central measurements that have low variation) that in turn was normalized by the autosomal median coverage. This operation resulted in a limited array of values (0, -0.5, -1, or -1.5) that were direct indicators of the copy number of the Y chromosome. The information contained in Supplementary Table 2 shows that median values of chromosomal coverage using WGS data are very stable measurements for all chromosomes. In contrast, values from the PCR-based method came from a single locus on the Y chromosome, and although the coverage values also depended on the copy number of the analyzed gene, they have larger variations due to stochasticity during the PCR, product purification, and/or sequencing. As we explain in the Discussion, we recommend applying the PCR-based method as a general strategy to evaluate whether LOY is present or not in a species/tissue using medium/large sample sizes. But we discourage taking the values for individual samples from the PCR-based method as exact LOY estimates; more precise LOY estimates for specific samples should come from whole-genome sequencing data.

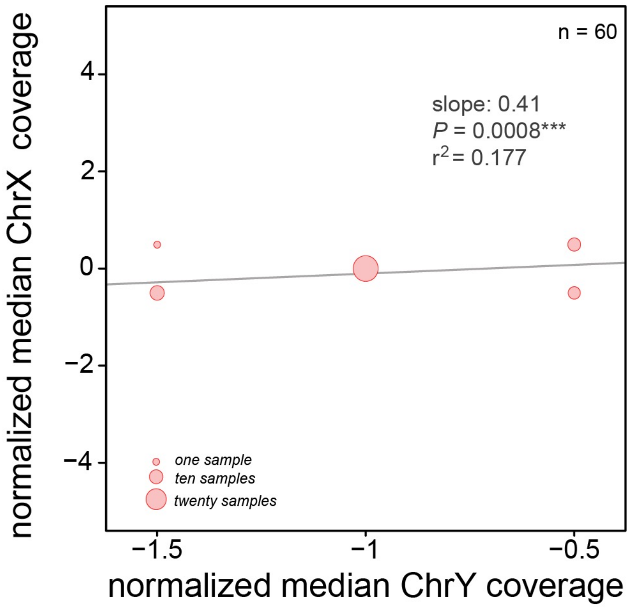

Supplementary Figure 3. Dot plot showing the normalized median sequencing coverage of the Y chromosome (ChrY) compared to the normalized median sequencing coverage of the X chromosome (ChrX) using the re-sequencing data. The coverage of the X and Y chromosomes was standardized using an expected value calculated as half of the median autosomal coverage (both sex chromosomes are single copy in the male genome). Significant differences, Linear Model: lm[median.chrX.coverage ~ median.chrY.coverage], *** indicates P < 0.001.

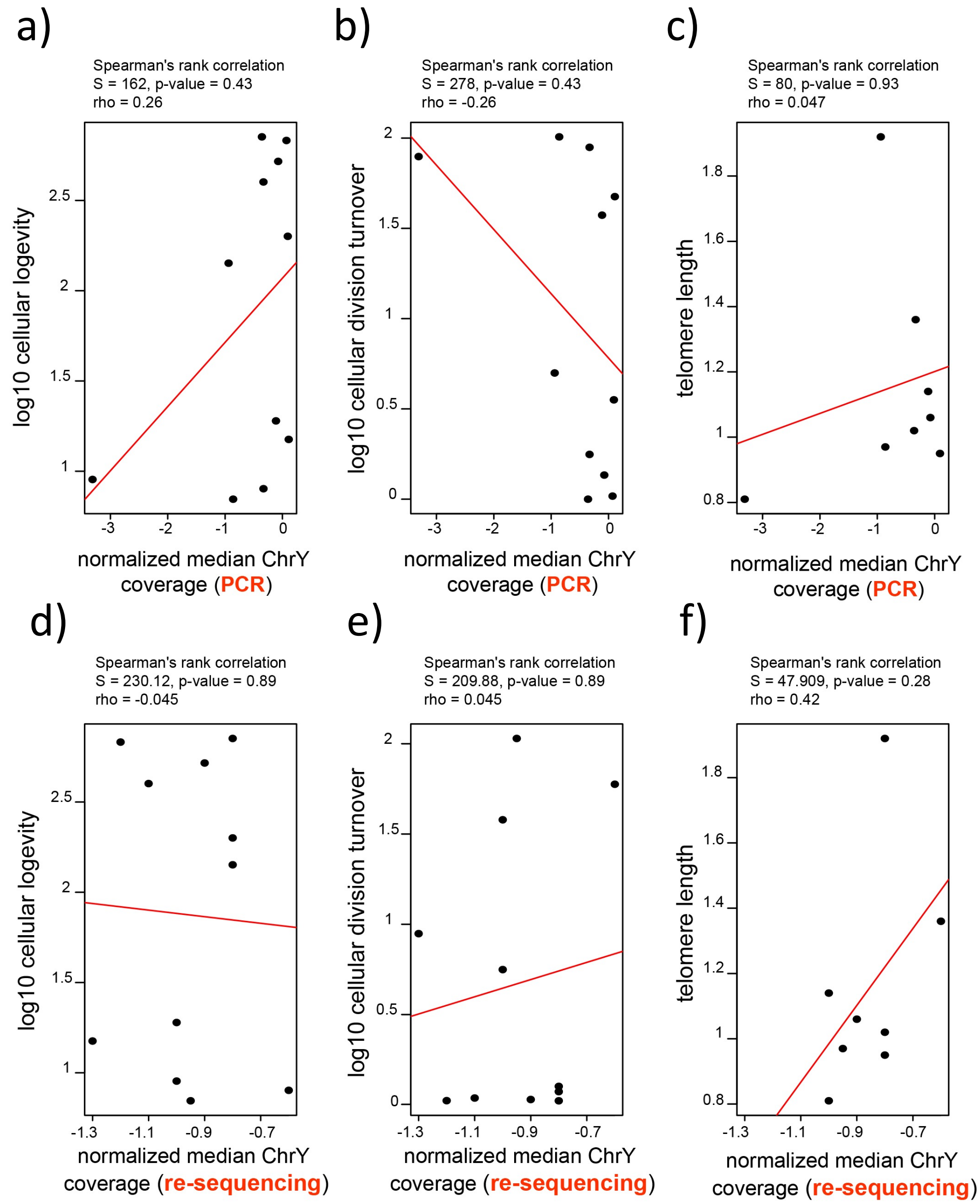

Supplementary Figure 4. Correlations between cellular longevity, cellular division turnover, and the length of telomers against the normalized coverage of the Y chromosome obtained from the PCR-based method (a-c) or using the re-sequencing data (d-f). Values and references for cellular longevity, cellular division turnover, and the length of telomers in eleven tissues of the rat are presented in Supplementary Table 3.

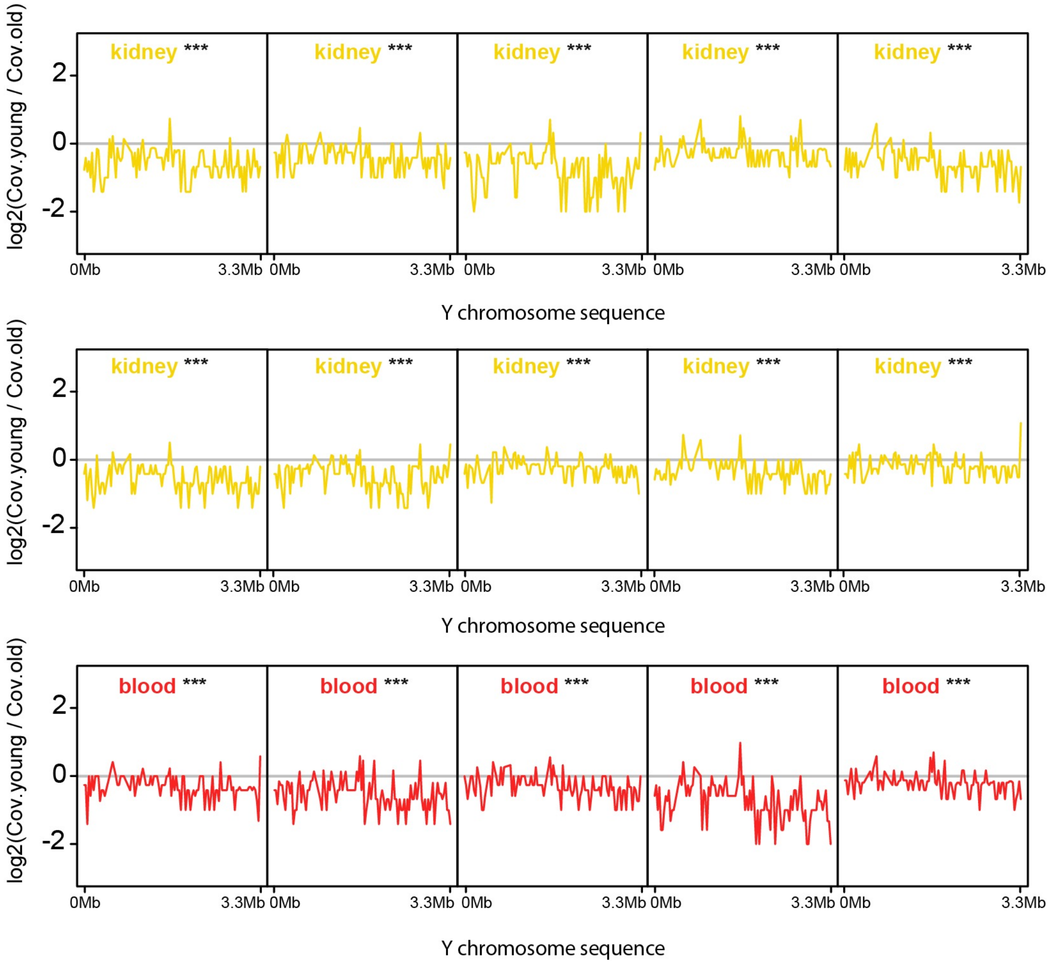

Supplementary Figure 5. Log_2 ratios of the read coverage in the young rat (Cov.young) divided by the read coverage in old rats (Cov.old) using non-overlapping sliding windows of 100kb over the first 3.3Mb of the Y chromosome sequence. The grey line indicates a log_2 ratio of zero, which represents an identical coverage between the young and old rats. Log_2 ratios above zero may indicate duplicated regions in the re-sequenced genomes that are not reported in the reference genomic sequence. Log_2 ratios below zero may indicate loss of the Y chromosome in the re-sequenced genomes. Ten samples from kidney are shown in yellow, and five samples from blood are shown in red. Significant differences, Mann–Whitney U test against a distribution with a fixed median of 0 (similar read coverage between the young and old samples). P-values were Benjamin–Hochberg corrected. *** indicates $P < 0.001$.

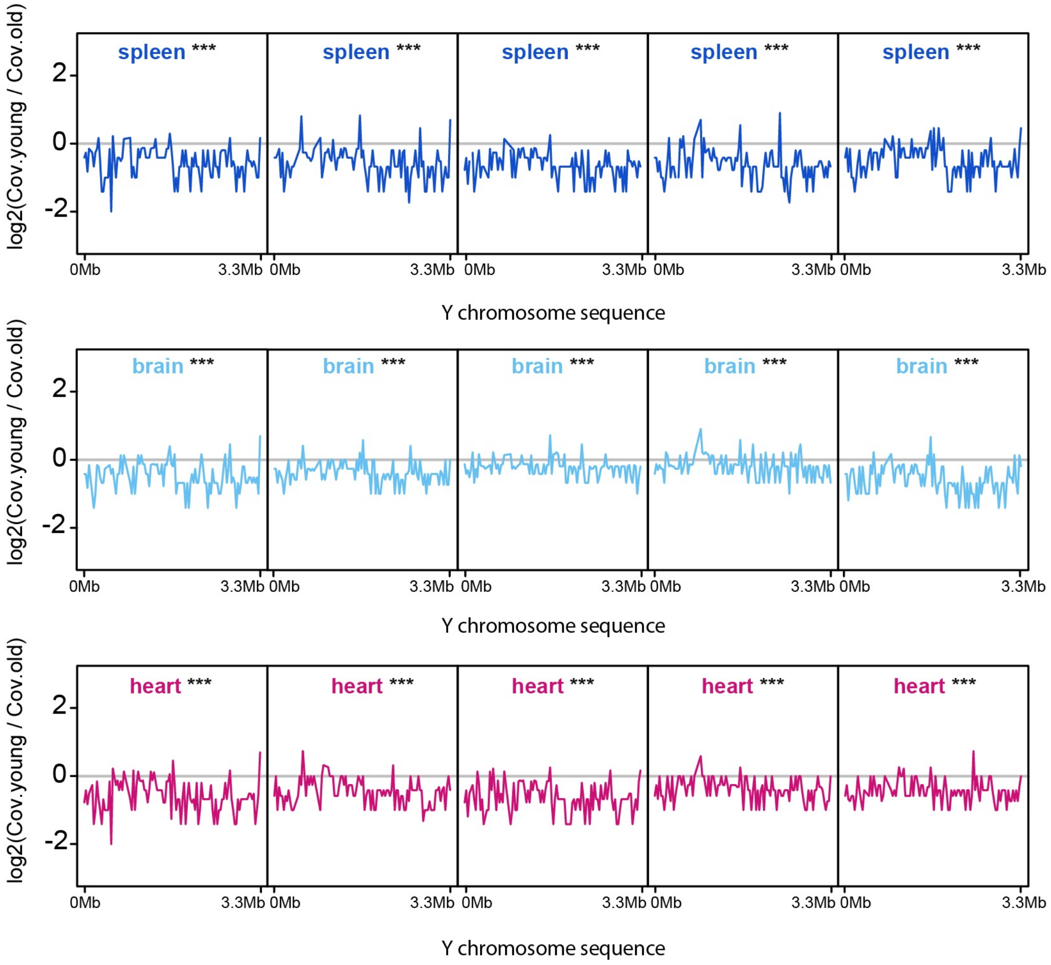

Supplementary Figure 6. Log_2 ratios of the read coverage in the young rat (Cov.young) divided by the read coverage in old rats (Cov.old) using non-overlapping sliding windows of 100kb over the first 3.3Mb of the Y chromosome sequence. The grey line indicates a log_2 ratio of zero, which represents an identical coverage between the young and old rats. Log_2 ratios above zero may indicate duplicated regions in the re-sequenced genomes that are not reported in the reference genomic sequence. Log_2 ratios below zero may indicate loss of the Y chromosome in the re-sequenced genomes. Five samples from spleen are shown in dark blue. Five samples from brain are shown in light blue. Five samples from heart are shown in dark pink. Significant differences, Mann–Whitney U test against a distribution with a fixed median of 0 (similar read coverage between the young and old samples). P-values were Benjamin–Hochberg corrected. *** indicates $P < 0.001$.

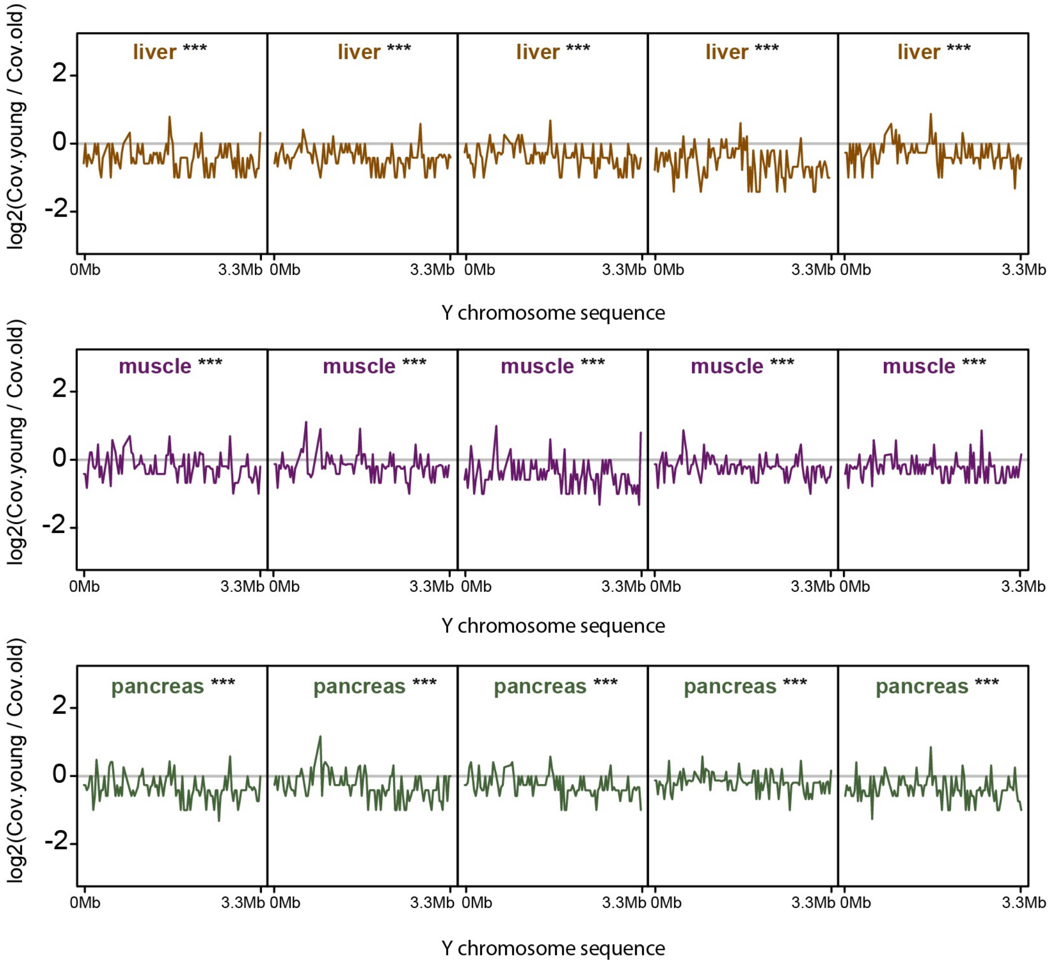

Supplementary Figure 7. Log_2 ratios of the read coverage in the young rat (Cov.young) divided by the read coverage in old rats (Cov.old) using non-overlapping sliding windows of 100kb over the first 3.3Mb of the Y chromosome sequence. The grey line indicates a log_2 ratio of zero, which represents an identical coverage between the young and old rats. Log_2 ratios above zero may indicate duplicated regions in the re-sequenced genomes that are not reported in the reference genomic sequence. Log_2 ratios below zero may indicate loss of the Y chromosome in the re-sequenced genomes. Five samples from liver are shown in brown. Five samples from muscle are shown in purple. Five samples from pancreas are shown in dark green. Significant differences, Mann–Whitney U test against a distribution with a fixed median of 0 (similar read coverage between the young and old samples). P-values were Benjamin–Hochberg corrected. *** indicates $P < 0.001$.

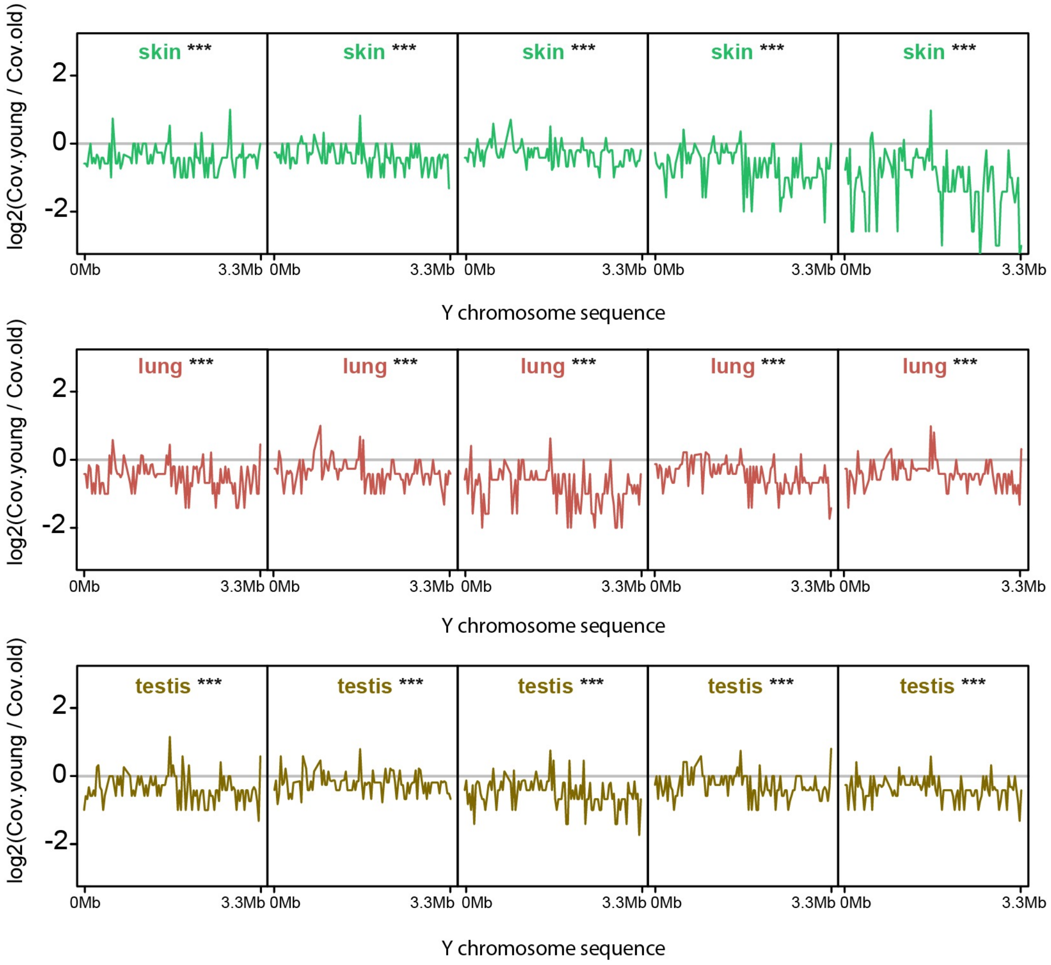

Supplementary Figure 8. Log_2 ratios of the read coverage in the young rat (Cov.young) divided by the read coverage in old rats (Cov.old) using non-overlapping sliding windows of 100kb over the first 3.3Mb of the Y chromosome sequence. The grey line indicates a log_2 ratio of zero, which represents an identical coverage between the young and old rats. Log_2 ratios above zero may indicate duplicated regions in the re-sequenced genomes that are not reported in the reference genomic sequence. Log_2 ratios below zero may indicate loss of the Y chromosome in the re-sequenced genomes. Five samples from skin are shown in light green. Five samples from lung are shown in light brown. Five samples from testis are shown in dark yellow. Significant differences, Mann–Whitney U test against a distribution with a fixed median of 0 (similar read coverage between the young and old samples). P-values were Benjamin–Hochberg corrected. *** indicates $P < 0.001$.

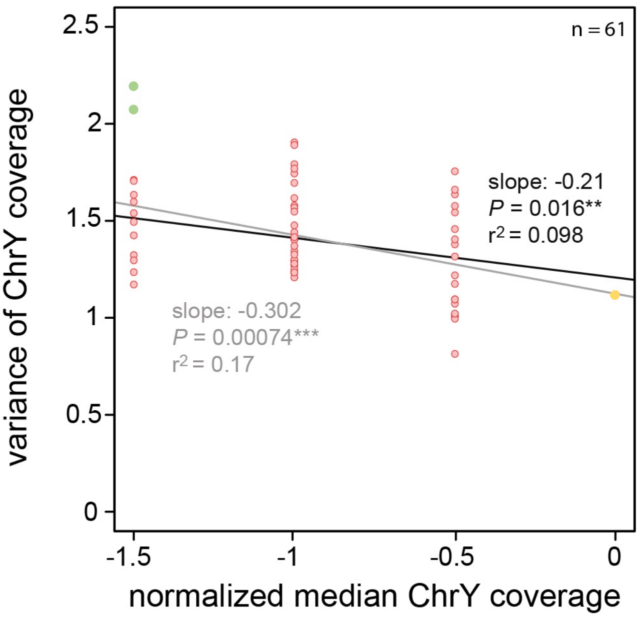

Supplementary Figure 9. Dot plot showing the median values of the normalized coverage of the Y chromosome (ChrY) against the variance of the coverage over the Y chromosomal sequence. Significant differences, Linear Model: lm[variance.coverage ~ median.chrY.coverage]; ** indicates $P < 0.01$; *** indicates $P < 0.001$. The results from two linear models are shown: the linear tendency in light grey includes all data points, whereas the linear tendency in black excluded the yellow dot (young ChrY) and the two green dots (old samples with the largest variance).
