# Supplementary material for: Rats exhibit age-related mosaic loss of chromosome Y
Source: Commun Biol. 2021 Dec 21;4:1418. doi: 10.1038/s42003-021-02936-y (PMC8692441; doi:10.1038/s42003-021-02936-y)
Supplement: Supplementary file 2 — Description of Additional Supplementary Files [file 42003_2021_2936_MOESM2_ESM.pdf]

Description of Additional Supplementary Files

File name: Supplementary Data 1-3
Description:

Supplementary Data 1. Data for cell division, cell longevity, and length of the telomeres in eleven tissues of rat.

Supplementary Data 2. Data from the 339 samples analyzed with the PCR-based approach.

Supplementary Data 3. Data from the 60 re-sequenced genomes.
